# 1105. Case Study. Topical Use of Erythropoietin to Promote Healing in Widely Meshed Skin Grafts-Case Report

**DOI:** 10.1093/jbcr/irag033.209

**Published:** 2026-04-06

**Authors:** Maysa Shemmiyeva, Alan Pang, Aarthi Annamalai, Emeka Odukwu

**Affiliations:** Texas Tech University Health Sciences Center, Lubbock, TX; Texas Tech University Health Sciences Center, Lubbock, TX; Texas Tech University Health Sciences Center, Lubbock, TX; Texas Tech University Health Sciences Center, Lubbock, TX

## Abstract

**Patient Presentation (age range, injury details, relevant history):**

A 42-year-old previously healthy male sustained 50% TBSA flame burns involving the face, torso, back, and bilateral upper extremities during a residential fire. He was intubated on arrival for grade 3 inhalation injury confirmed by bronchoscopy. Hospital course included tangential excision and 4:1 meshed split-thickness skin grafting (STSG) of the back with allograft overlay.

**Clinical Challenges:**

Despite optimal wound care and negative tissue cultures, the interstices of the 4:1 mesh remained unhealed more than three weeks post-grafting. Prolonged open interstices increased risk of infection, delayed rehabilitation, and threatened graft success.

**Management Approach:**

On postoperative day 21, topical epoetin alfa-epbx (Epogen 10 000 U/mL) was mixed with a non-adherent neomycin dressing and applied once daily for three consecutive days. The goal was to stimulate epithelialization and promote bridging of non-healed interstices. Nutritional status and systemic labs were monitored and remained normal.

**Outcomes:**

By day 4 post-treatment, there was marked reduction in open interstices with visible epithelial bridging. No local infection, allergic reaction, or systemic adverse effects were observed. The patient’s wounds continued to epithelialize, and he was discharged with favorable prognosis and minimal expected scarring.

**Lessons Learned:**

Topical erythropoietin may enhance angiogenesis and epithelial migration in widely meshed STSGs when standard wound care fails to achieve closure. Its use was safe, well-tolerated, and produced rapid clinical improvement without complications.

**Applicability to Practice:**

This case highlights topical erythropoietin as a potential adjunct for delayed interstice healing, offering burn surgeons a low-risk, readily available option to accelerate epithelialization, reduce infection risk, and shorten hospitalization. Controlled trials are warranted to define dosing, frequency, and patient selection.

